# Systematic review on the prevalence of illness and stress and their associated risk factors among educators in Malaysia

**DOI:** 10.1371/journal.pone.0217430

**Published:** 2019-05-28

**Authors:** Kwee Ling Tai, Yee Guan Ng, Poh Ying Lim

**Affiliations:** 1 Department of Environmental and Occupational Health, Faculty Medicine and Health Sciences, Universiti Putra Malaysia, Serdang, Selangor Darul Ehsan, Malaysia; 2 Department of Community Health, Faculty Medicine and Health Sciences, Universiti Putra Malaysia, Serdang, Selangor Darul Ehsan, Malaysia; Gazi University, Faculty of Health Sciences, TURKEY

## Abstract

**Background:**

Despite evidence of physical (illness) and mental (stress) health problems, there appears to be a lack of studies or concern regarding occupational safety and health among educators in Malaysia.

**Objective:**

To review the prevalence of illness, stress, and corresponding risk factors among educators in Malaysia.

**Method:**

Scopus, ProQuest, PubMed, ScienceDirect, CAB, and other computerized databases were searched according to Preferred Reporting Items for Systematic Reviews and Meta-Analyses (PRISMA) guidelines to identify studies published between January 2013 and April 2019 on the prevalence and associated risk factors of illness and stress among educators (S1 Checklist). The keywords used included educator, teacher, lecturer, academic staff, teaching profession, university staff, academician, faculty, illness, injury, disease, pain, WMSD, dysphonia, hoarseness, stress, mental health, strain, health problem, disorder, and/or Malaysia. Selected studies were evaluated by quality assessment.

**Results:**

Twenty-two articles fulfilled the eligibility criteria. The prevalence of illness and stress was determined for low back pain (33.3–72.9%); upper back pain (33.33–56.4%); neck/shoulder pain (40.4–80.1%); upper arm discomfort (91.3%); forearm pain (89.6%); wrist pain (16.7–93.2%); hip pain (13.2–40.9%); thigh discomfort (91.8%); lower leg discomfort (90.5%); knee pain (23.7–88.0%); ankle/feet pain (19.3–87.7%); elbow pain (3.5–13.0%); voice disorder (10.4–13.0%) and stress (5.5–25.9%). Sex, education level, teaching experience, quality of life, anxiety, depression, coping styles, and others were reported as associated risk factors across the studies.

**Conclusions:**

There appears to be a cause for concern regarding musculoskeletal disorders, voice disorder, and stress reported among educators in Malaysia. While most risk factors matched those reported in studies elsewhere, others such as school characteristics (school level, government or private school, and location [rural/urban]) have not been investigated.

## Introduction

Occupational health (OH) has attracted increasing attention in Malaysia in recent years, including that in the academic sector. The International Labour Organization (ILO/WHO) (1950) defines OH as *“the promotion and maintenance of the highest degree of physical*, *mental and social well-being of workers in all occupations by preventing departures from health*, *controlling risks and the adaptation of work to people*, *and people to their jobs”* [1]. In the academic sector, educators play a pivotal role in ensuring that their learners achieve the expected learning outcomes corresponding to the level of education and educational policy. However, the quality of teaching and learning activities may be affected where educators become ill or injured due to occupational hazards in the workplace.

Various studies have indicated that OH problems in academic sectors can be divided into physical health problems (illnesses) and mental health problems (stress). Among the different types of illness such as voice, musculoskeletal, and upper limb disorders, low back pain (LBP), neck/shoulder pain (NSP), elbow pain, and wrist pain have been reported in recent studies by Akinbode, Lam, Ayres, & Sadhra (2014); Behlau, Zambon, Guerrieri, & Roy (2012); Karwan, Azuhairi, & Hayati (2015); Mohan, Justine, Jagannathan, Aminudin, & Johari (2015); Mohd Anuar, Rasdi, Saliluddin, & Zainal Abidin (2016); Rajan, Chellappan, & Thenmozhi (2016); Seifpanahi et al. (2016); Van Houtte, Claeys, Wuyts, & Van Lierde (2011); Zamri, Moy, & Hoe (2017) [2–10]. Other issues reported include abnormal lipid profiles [11], auditory problems [12], respiratory problems [13], and gastroesophageal reflux disease (GERD) [14].

Similarly, stress has been consistently reported as a common mental health problem among educators [15–29]. Stressed educators may exhibit negative outcomes that affect their students as stressed teachers may have unforeseen, extreme, or drastic negative responses and behaviors and become intolerant of students, thus affecting the quality of education provided to students [30]. Mukosolu et al. (2015) reported a prevalence of stress among local educators of 23.1%, which was higher than that among non-educators (19.8%).

In order to address OH issues among educators, risk factors have reported. The risk factors associated with the OH problems can be divided into two categories, socio-demographic factors and occupational factors. The socio-demographic factors include sex, age, marital status, and education level [12,15,31–34] whereas the occupational factors include school characteristics, teaching experience, workload, and teaching experience [31,32,35–41]. Investigating the associated risk factors of OH problems allow relevant authorities to plan and institute policies and preventive measures, thereby enabling educators to work in low-risk environments and, thus, provide better education to their students [42].

**Fig 1:**
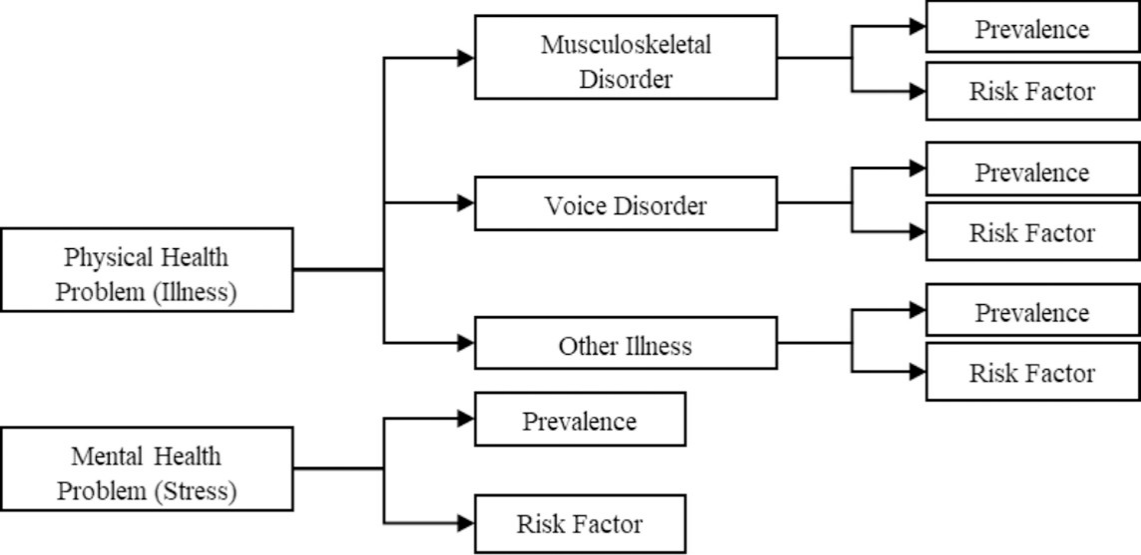
Framework of variables included in the review.

Therefore, the main objective of this study was to review (i) the prevalence of physical health problem (illnesses) and mental health problem (stress) among educators and (ii) the risk factors for both health problems among educators specifically in Malaysia. The framework of variables in this review is shown in Fig 1.

## Materials and methods

A systematic review was carried out to identify observational studies reporting on the prevalence and associated risk factors of illness and stress among educators in Malaysia that were published between January 2013 and April 2019. This systematic review was conducted using five electronic databases, including Scopus, ProQuest, PubMed, ScienceDirect, and CAB Direct.

### Eligibility criteria

This review included articles meeting the following criteria: [1] studies carried out in Malaysia; [2] published articles; [3] articles with available full text; [4] articles with an English version; [5] observational studies; [6] clear definition of educators as the study population and; [7] articles with the keywords detailed in Table 1.

**Table 1:** Keywords used for the literature search.

Educator	Teacher/ lecturer/ academic staff/ teaching profession/ university staff/ academician/ faculty	
**AND**		
Illness	Injury/ disease/ pain/ WMSD/ dysphonia/ hoarseness	**OR** Health problem/ disorder
**OR**		
Stress	Mental health/ strain	
**AND**		
Malaysia		

The keywords used were educators (teacher OR lecturer OR academic staff OR teaching profession OR university staff OR academician OR faculty) AND illness (injury OR disease OR pain OR work-related musculoskeletal disorder (WMSD) OR dysphonia OR hoarseness) AND stress (mental health OR strain) AND health problem (disorder) AND Malaysia, as shown in Table 1.

### Selection of literature (screening and eligibility)

Following a comprehensive literature search of five electronic databases, the relevant literature identified using the keywords in Table 1 were recorded. Studies which were not conducted in Malaysia, without available full texts, in languages other than English, and irrelevant studies were excluded from this review using the filtering tool of the database. From the remaining records, other additional articles which did not appear in the search engine databases were identified from the reference lists of each study. The list of literature which fulfilled the criteria was further screened by three reviewers based on the title, abstract, keywords, and statement in eligibility criteria. Studies with irrelevant information, duplicated publications, and review articles were removed.

**Fig 2:**
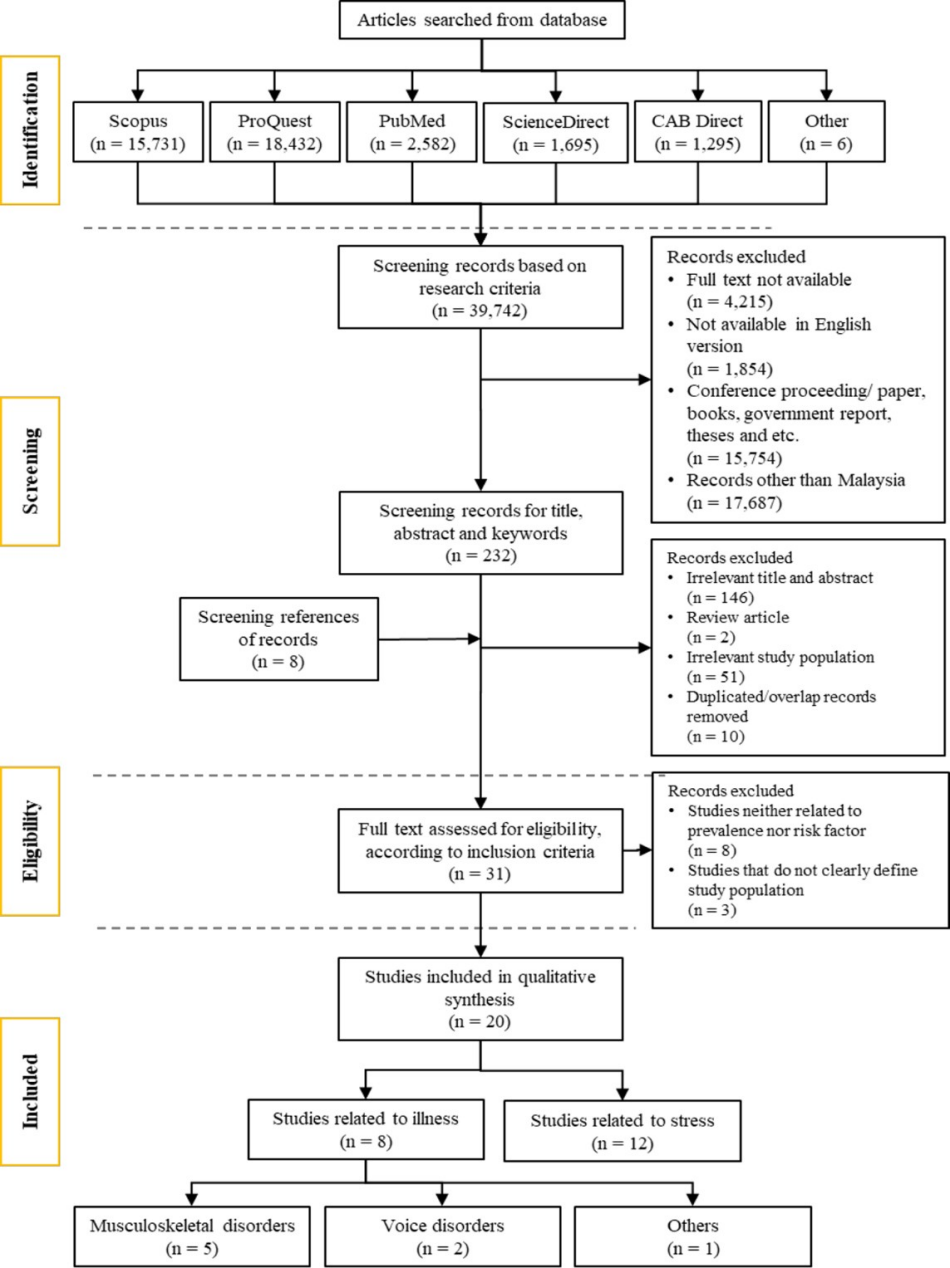
Flowchart of the review process.

As shown in Fig 2, out of 43,429 potential articles, 43,060 were excluded based on the inclusion and exclusion criteria. The remaining 369 articles were screened based on their titles, abstracts, and keywords. Among the 369 articles, 267 irrelevant, 10 review articles, 52 with irrelevant study populations, and 13 with duplicated records were excluded. This screening process yielded 35 articles which met the eligibility criteria. The relevant data of each study including the authors, year of the study, study population, sample size, methodology or instrument used, type of health problems, risk factors, and quantitative outcomes were recorded.

The remaining studies were then screened based on the full articles to ensure their eligibility. A total of 13 articles were further excluded as nine of them did not report prevalence or risk factors of OH problems and four articles did not clearly define the study population. A total of 22 studies fulfilled all eligibility criteria for this review. The article selection and extraction process were based on the Preferred Reporting Items for Systematic Reviews and Meta-Analyses (PRISMA) statement [43], as shown in Fig 2.

Out of 22 studies among educators, 10 and 12 were related to illness and stress, respectively. Fifteen of the 22 studies reported both the prevalence and risk factors of physical or mental health problems among educators while seven reported only the risk factors. Due to the use of different instruments and analytic tools in the selected studies, a meta-analysis could not be conducted for this systematic review.

### Quality assessment of the literature

A quality assessment was carried out by the researchers on the selected articles using the modified assessment tools as described by Ibrahim et al. (2013), which were based on those in the study by Parker et al. (2008) for epidemiology prevalence studies [44]. The quality assessment tool consisted of seven items (quality markers), as follows: [1] sample definition; [2] recruitment; [3] representative sample; [4] response rate; [5] scale; [6] sample size; and [7] confidence interval (CI) or sample error (SE).

One point was scored for each fulfilled quality marker, for a maximum total score of 7 using this quality assessment tool. The descriptive criteria for each of quality marker were clearly defined based on the study by Ibrahim et al. (2013), as shown in Table 2, whereas the results of the quality assessment for each of the selected studies are shown in S1 Appendix.

**Table 2:** Description of the quality markers.

Item	Quality Marker	Description
1	Sample definition	The target population was defined clearly.
2	Recruitment	Complete, random or consecutive recruitment (sampling).
3	Representative sample	The targeted sample is representative, or the report presents evidence that the results can be generalized to the educators.
4	Response rate	The response rate was equal or greater than 70%.
5	Scale	The scale used is a validated measure of illness and stress (validated instrument).
6	Sample size	The sample size is adequate with a minimum sample size of 300.
7	Confidence intervals (CI) or sample error (SE)	The confidence intervals (CI) and sample error (SE) are reported.

Taken from: Ibrahim AK, Kelly SJ, Adams CE, Glazebrook C. A Systematic Review of Studies of Depression Prevalence in University Students. Journal of Psychiatric Research [Internet]. 2013;47(3):391–400. Available from: http://dx.doi.org/10.1016/j.jpsychires.2012.11.015

## Results

In the 22 articles reviewed, the studied population (educators) were from different levels of school in Malaysia. Three articles studied primary school teachers, six included secondary school teachers, 11 assessed lecturers or academicians at higher learning institutions, and two articles did not clearly specify the level of schools from which the educators were recruited (Table 3).

**Table 3:** Summary of the selected local studies.

Illness/Stress	Author & Year	Study Population & Sample Size	Methodology/ Instrument	Prevalence Rates (%; for teachers only); 95% CI	Risk Factors			
					Socio-demographic	Occupational	Psychosocial	Others
Musculoskeletal Disorder	Karwan et al. (2015)	Public university workers: Educators (155) Non-educators (116)	Nordic Questionnaire	Upper limb disorder (67.2%): Neck pain (54.2%); Shoulder pain (47.2%); Elbow pain (13%); Wrist/hand pain (28.1%)	Age, body mass index (BMI)	Duration of employment	‒	Smoking, exercise
	Mohan et al. (2015)	Academicians: Educators (228) Non-educators (none)	Extended Nordic Musculoskeletal Questionnaire (NMQ-E) for symptoms and Dutch Musculoskeletal Questionnaire (DMQ) for risk factors	Neck pain (44.7%); Shoulder pain (40.4%); Upper back pain (33.3%); Low back pain (33.3%); Elbow pain (3.5%); Wrist pain (16.7%); Hip pain (13.2%); Knee pain (23.7%) Ankle/ feet pain (19.3%)	Sex	Physical posture	‒	‒
	Mohd Anuar et al. (2016)	Secondary school teachers: Educators (30) Non-educators (none)	Nordic Questionnaire for symptoms and DMQ for risk factors	Low back pain (72.9%)	Sex	Prolonged sitting, walking up and down stairs, lifting loads with hands	‒	‒
	Rajan et al. (2016)	Secondary school teacher Educator (260) Non-educator (none)	Nordic questionnaire	Low back pain (62.5%)	‒	Prolonged standing, prolonged sitting, working with computers	‒	‒
	Zamri et al. (2017)	Public school secondary school teachers: Educators (1,482) Non-educator (none)	Nordic Musculoskeletal Questionnaire (NMQ) for symptoms and Depression Anxiety Stress Scale (DASS 21) for psychological factors	Low back pain (48.0%, (45.2–50.9); Neck/shoulder pain (60.1%, 57.4–62.9); Stress (19.6%)	‒	‒	Low back pain (job demands, skill discretion); Neck/shoulder pain (supervisory support)	Low back pain (depression, anxiety, mental health); Neck/shoulder pain (anxiety, mental health)
	Sugumaran et al. (2019)	Academic staff: Educators (132) Non-educators (none)	-	Neck pain (41.0%)	-	-	-	Work-related factors, individual factors, work tension factors
	Ng et al. (2019)	Primary school teachers: Educators (367) Non-educators (none)	Work Organization Assessment Questionnaire (WOAQ) for workplace psychosocial hazards, Beck Depression Inventory for Malays (BDI-M) for depressive symptoms, and Cornell Musculoskeletal Disorder Questionnaire (CMSD) for musculoskeletal discomfort	Musculoskeletal disorders (80.1%, 75.8–84.2); Neck (75.5%); Shoulder (80.1%); Upper back (56.4%); Upper arm (91.3%); Low back (59.9%); Forearm (89.6%); Wrist (93.2%); Hip/buttocks (40.9%); Thigh (91.8%); Knee (88.0%); Lower leg (90.5%); Foot (87.7%)	-	-	Psychosocial factors (role conflict, job control, safety-specific leadership)	Depression
Voice Disorder	Moy et al. (2015)	Secondary school teacher Educator (6039) Non-educator (none)	Voice Handicap Index (VHI)	10.4% (7.1–14.9)	‒	‒	‒	Quality of life
	Roscellalnja (2016)	Full-time primary school teacher Educator (100) Non-educator (none)	Self-constructed questionnaire	13.0%	‒	‒	Teaching session	Smoking, consuming alcohol beverages
Abnormal Lipid profile (TC, TG, HDL, and LDL)	Ariaratnam et al. (2017)	Staff from the tertiary education center: Educators (78) Non-educators (117)	Coping Inventory for Stressful Situations (CISS) and National Cholesterol Education Program (NCEP) for lipid profiles	Only showed the odds ratio	Sex, age, education level	‒	‒	Avoidance-oriented coping styles
Stress	Azizah et al. (2016)	Private university’s lecturer Educator (113) Non-educator (none)	Job Content Questionnaire (JCQ)	24.8%	Sex, level of education	Income, job title, length of service	‒	‒
	Chen et al. (2014)	Private university academicians: Educators: (229) Non-educators: (none)	Maslach Burnout Inventory-Educators Survey (MBI-ES)	Burnout (5.5%)	‒	‒	‒	Quality of life
	Ismail et al. (2014)	Community college lecturers: Educators: (189) Non-educators: (none)	JCQ	25.9%	‒	‒	Psychological job demands, decision latitude, social support, job insecurity	‒
	Mukosolu et al. (2015)	Universiti Putra Malaysia staff: Educators (294) Non-educators (217)	JCQ	21.7%	‒	Job demands	Social support	Depression, anxiety, coping strategies.
	Noor & Ismail (2016)	Research university’s academic staff Educator (380) Non-educator (none)	Depression Anxiety Stress Scale (DASS) for symptoms and Stress Sources Questionnaire (SSQ) for stressors	22.1%	Ethnic group	Teaching, research	Career development	‒
	Yaacob & Choi (2015)	Teachers Educator (386) Non-educator (none)	Occupational Role Questionnaire (ORQ) and Occupational Stress Indicator (OSI)	‒	‒	‒	‒	Job satisfaction
	Wee & Bahrein (2016)	Secondary school teacher Educator (300) Non-educator (none)	Teachers’ Attribution of Responsibility for Stress Questionnaire (TARSQ)	‒	‒	‒	‒	Emotional intelligence
	Nor & Salleh (2015)	Primary school teachers: Educators: (10) Non-educators: (none)	Electroencephalograms (EEG) machine and DASS	‒	‒	‒	‒	Precursor emotion
	Ahmad et al. (2015)	Secondary school counselors: Educators (205) Non-educators (none)	Tennessee Self-Concept Scale (TSCS) and Teacher Stress Inventory	‒	‒	Income	Supervision, promotion	Self-concept
	Ismail et al. (2013)	Academic staff: Educators (320) Non-educators (none)	A self-constructed questionnaire based on job stress literature	‒	‒	‒	Supervisor’s support	
	Hamjah et al. (2015)	University academicians: Educators (37) Non-educators (none)	Self-constructed questionnaire	‒	Religion (spiritual approach)	Workload	‒	‒
	Ghani et al. (2014)	Special education teachers: Educators (92) Non-educators (none)	Teacher Stress Inventory	‒	‒	Workload	Student misbehavior, resources difficulties, recognition, interpersonal relationships	‒

The quality assessment scores of the 22 articles ranged from 2 to 7, with a mean of 4.50 (SD = 1.22). The overall sample size in this review was 11,876 respondents, with a minimum sample of 10 and a maximum of 6,039. Respondent sex and age were both reported in all but six of the selected [11,19,20,23,28,29]. Women made up the majority of respondents in all except five studies [18–20,28,45]. Only three of the selected articles had response rates below 75% [7,10,18]; the other 19 studies had rates above 75% [2,8,9,11,15,16,19,20,22–25,27–29,45–48]. The prevalence and/or risk factors of 22 selected studies are summarized in Table 3.

### Prevalence of illness and stress

Among the various types of illnesses, the prevalence of musculoskeletal disorders and voice disorders among local educators were most commonly reported. The prevalence of low back pain (LBP) was 33.3–72.9%; upper back pain (UBP), 33.3–56.4%; neck/shoulder pain (NSP), 40.4–80.1%; upper arm discomfort, 91.3%; forearm discomfort, 89.6%; wrist pain, 16.7–93.2%; hip pain, 13.2–40.9%; thigh discomfort, 91.8%; lower leg region discomfort, 90.5%; knee pain, 23.7–88.0%; ankle/feet pain, 19.3–87.7%, and elbow pain, 3.5–13.0% [2,7–10,45,48]. The prevalence of voice disorders ranged from 10.4–13.0% [46,47].

The prevalence of stress was only reported among educators from higher learning institutions (colleges and universities), ranging from 5.5% to 25.9% with 5.5% reporting burnout among academicians in private universities [15,16,22,24,25]. None of the other studies reported the prevalence of stress among primary and secondary school educators in Malaysia.

### Risk factors for illness

In terms of socio-demographic risk factors, three articles demonstrated a significant association of sex, age, education level, and Body Mass Index (BMI) with musculoskeletal disorders, voice disorder, and abnormal lipid profiles. Women reported a higher prevalence of musculoskeletal disorders (neck, shoulder, low back, upper back, elbow pain, etc.) compared to that in men [7,8]. However, men had a higher prevalence of abnormal lipid profiles compared to that in women [11].

**Fig 3:**
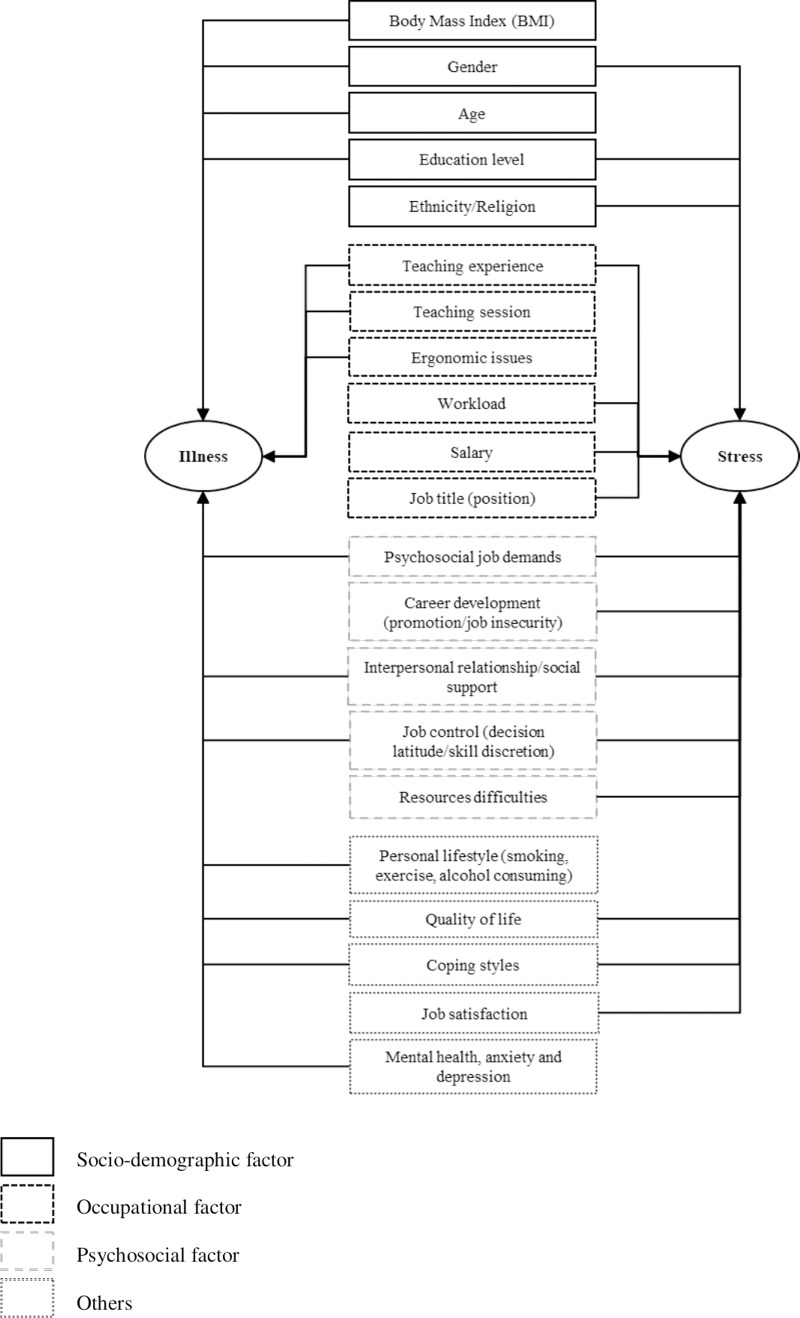
Associated risk factors of illness and stress.

Educators with older age reported more musculoskeletal disorders and abnormal lipid profiles compared to those in younger educators [2,11]. Educators with higher education levels had a lower prevalence of abnormal lipid profiles than those in educators with lower levels of education [11]. Obese educators with high BMI had a higher prevalence of upper limb disorders than that in educators who were not obese [2]. Fig 3 shows the associated risk factors of illness and stress among local educators.

In terms of occupational factors, three articles showed the association between the prevalence of musculoskeletal and voice disorders with teaching experience (duration of employment) and teaching sessions. The prevalence of musculoskeletal disorders was positively associated with longer teaching experience (duration of employment) [2]. Teaching session (morning or evening) was associated with voice disorders, in which educators in morning sessions had a higher prevalence of voice disorders [47]. Educators exposed to ergonomic risk factors including prolonged standing, prolonged sitting, working with computers, walking up and down stairs, and working with loads had with a higher prevalence of musculoskeletal disorders [7–9].

Personal lifestyle behaviors such as smoking, alcohol consumption, and low frequency of exercise are reported risk factors for musculoskeletal and voice disorders [2,47]. Others risk factors of musculoskeletal disorders (lower back and neck/shoulder pain) include poor mental health, psychological factors (depression and anxiety), and psychosocial factors (low skill discretion, low supervisory support, role conflict, job control, and safety-specific leadership) [10,48]. Avoidance-oriented coping styles were negatively associated with a lower prevalence of abnormal lipid profiles [11].

### Risk factors for stress

The review of the articles identified sex, education level, ethnicity, and religion as risk factors for stress among educators in higher learning institutions in Malaysia. Men had a higher prevalence of stress compared to that in women whereas lower education level was associated with lower stress [24]. Comparing between Malays and non-Malays (ethnicity), Noor & Ismail (2016) reported that the latter had a higher prevalence of stress than the former, findings contrary to those in a study by Mukosolu et al. (2015). Educators with strong spiritual practices also showed lower stress levels [19].

High or heavy workloads and job demands among educators were positively associated with stress [16,19,20,22]. Career development (getting to a higher position), research work, and teaching had a positive relationship with the level of stress among educators [15,29]. The prevalence of stress was also influenced by salary, in which educators with a low salary (pay benefit or income) reported a higher prevalence of stress [24,29].

Educators with longer teaching experiences and with high social support (co-worker and supervisor support or supervision) reported a lower prevalence of stress compared to the prevalence in educators without these factors those; namely, shorter service rendered [24] and without or low social support [16,22]. In term of job title, which is relative to functions and roles, assistant lecturers reported a higher prevalence of stress than that in professors and lecturers [24].

The precursor emotion of educators reflected their current stress status [28]. Poor decision latitude, lack of job insecurity, and job dissatisfaction were significantly and positively associated with stress [16,23]. Student misbehavior, time and resource difficulties, lack of recognition (as reported by administrators), poor interpersonal relationships, and avoidance focused coping styles (focus and venting of emotion and self-blame) were a source of stress among educators [20,22].

## Discussion

Similar to most education tier systems worldwide, teachers or educators in Malaysia can be generally classified as pre-school, primary school, secondary/high school, and university and college (which usually uses the term academician to reflect the research components in the higher learning institutions) educators. The results of this review illustrate the apparent lack of study on a homogenous group of educators regarding a particular health effect outcome or associated risk factors in Malaysia except for stress.

For example, despite the harmonious use of the (modified) Nordic Musculoskeletal Questionnaire (NMQ) as the base instrument for determining the prevalence of musculoskeletal disorders (MSDs) in the studies found in this review, the study populations differed. Among those reviewed, three studies among secondary school teachers focused primarily on low back pain reported considerable differences in prevalence [8–10], whereas the prevalence of MSDs among academicians or primary school teachers were reported comprehensively in three studies [2,7,48].

Compared to studies in several other developing countries [49], the prevalence of MSDs varied. Using low back pain as an example, a study in Iran reported a prevalence of 31.1% [50], compared to 45.6% in China [51], 55.7% in Botswana [52], 57.5% in Ethiopia, [53], 68.8% in Taiwan (a province of China) [35], 63.8% and 66.9% in Saudi Arabia in two separate studies [54,55]. The prevalence was highest in Pakistan, at 87.0% [56], whereas the prevalence in Norway, a developed country, was 29.1% [57].

The prevalence of voice disorders and the risk factors could not be compared between the two studies on this topic as the instruments (questionnaire) differed. As voice is a primary asset for the delivery of services to their learner, voice disorders have been widely reported by studies in Brazil (17.1–39.6%) [58–60], India (17.4%) [61], Belgium (51.2%) [6], and Iran (54.6%) [3].

The differences in the prevalence of voice disorders between studies could be attributable to the inconsistent choice of words to describe voice disorders between local and non-local studies. For example, Akinbode et al. (2014) and da Rocha et al. (2015) reported dysphonia as a voice disorder, while Korn et al. (2015) used the word hoarseness and studies in Malaysia concurrently used dysphonia, hoarseness, and other voice-related problems to ensure comprehensive coverage and understanding.

Workplace stress has been a long-standing mental health problem and a priority of the International Labour Organization. The results of this review indicated that stress was addressed by a significantly higher number of studies compared to other illnesses. Amongst studies in Malaysia, only those studies involving academicians reported a consistent prevalence of stress (21.7–25.9% among four studies) and corresponding risk factors, whereas the studies of school teachers (both primary and secondary) only investigated and reported the risk factors of stress but did not report their prevalence.

The prevalence of stress among academicians in Malaysia cannot be directly compared with the prevalence reported in Germany (18%) [41], which was performed in a developed country and included only female teachers. On the other hand, the different prevalence of stress in Iran, another developing country (39.5%) [62] could be attributable to differences in cultures and education systems [15].

In terms of risk factors, the studies in this review revealed a range of different risk factors for MSDs in different categories. Between the three studies among academicians, socio-demographic, occupational, and other risk factors did not show any similarities [2,7,45]. However, among secondary school teachers, prolonged sitting was reported by two of three studies [8,9]. Sex was also a risk factor for both academicians and secondary school teachers in two separate studies [7,8].

The socio-demographic risk factors identified in this review were in line with common findings. Previous studies in similar populations in other countries also found that female sex [52,53,63], increasing age [50,53,54,63,64] and obesity [50] were associated with a higher prevalence of MSDs than the contrary (male, younger, and non-obese).

As the role of educators is typically undertaken by women in Malaysia, the high participation rate of women in this profession potentially contributes to the high prevalence [50], whereas other studies suggested that low nutritional and overweight status places women at higher risk for MSDs [52,53]. Higher job stress or emotional exhaustion were also reported to cause a higher prevalence of MSDs among women [51,63], in addition to obesity [2].

In terms of age, the higher prevalence of MSDs among educators with longer teaching experiences could be due to natural age-related degeneration of the musculoskeletal system and correspondingly longer exposure to ergonomic risk factors at work [2,35,50,54,63]. Nevertheless, similar findings were not reported by Abdulmonem et al. (2014), Mohan et al. (2015), or Yue et al. (2012).

The ergonomic risk factors (particularly awkward posture, prolonged sitting and standing, lifting of loads, and walking up and down stairs) reported by the studies in this review were also similarly reported by studies in other countries on the similar populations [51,52,64]. However, regular exercise has been shown to relieve educator pain intensity and, thus, reduce the development of MSDs [52,63].

The various risk factors of stress reported by the studies in this review appear concordant with the notion proposed by the European Agency for Safety and Health at Work [65] that educators participate in a naturally stressful occupation. Our results indicated that there were little to no similarities in terms of the different categories of risk factors for stress within and between the different categories of educators (academicians vs. school teachers). Only several studies in this review investigated the effects of socio-demographic risk factors on stress in school teachers. Specifically, sex, education level, and ethnicity were the risk factors for stress among academicians.

Among occupational risk factors, income and salary were the common risk factors for stress across both groups of educators in two studies. Although further review found that school teachers with a higher salary or income had a lower prevalence of stress than that in those with a lower salary, a study in India concluded that teachers with higher salaries experienced higher stress [36]. The contrary findings in India were concordant with those of a study by Azizah et al. (2016) in which ambitious educators had higher expectations as they worked their way to higher positions or ranks (career development). In addition, school teachers with heavier workloads have a higher prevalence of stress attributable to their exhaustive work burdens [19,41].

Psychosocial factors such as increased role conflict, decreased job control, and lack of supervision can lead to the development of MSDs [48]. Supervisor social support and supervision from the leader at work can help to mitigate the effect on family conflict due to role ambiguity and work intrusion and, thus, reduce stress level among educators [18,22,29]. Furthermore, selected studies showed that educators’ stress was negatively associated with emotional intelligence or coping styles and good quality of life [22,25,27], as these educators were able to deal with their stress.

Only one study found in this review reported abnormal lipid profiles among academicians. An in-depth review indicated that older male educators and those with lower education level and without coping styles had a higher prevalence of abnormal lipid profiles. One plausible reason could be the degeneration of the digestive system (liver) among older educators and that women naturally have a higher percentage of fat [66]. Nevertheless, better-educated academicians may have a better understanding of healthy lifestyles and coping mechanisms and avoid consuming unhealthy foods and lead healthy lifestyles.

Overall, there appears to be lack of a general review of health conditions among educators in Malaysia. Studies on similar population outside of Malaysia have shown that besides MSDs, voice disorders, and stress, which were primarily reported in this review, other illnesses such as gastrointestinal disorder [14,57], allergies [67], and auditory problem [12] were also common among educators.

The risk factors of illnesses and stress found by studies in this review were generally similar to various other studies. However, there appears to be a lack of study amongst school teachers on occupational risk factors. School characteristics such as school level (primary, secondary, and tertiary), education system (government/public and private), school location (urban, suburban, and rural), and school type have been shown to affect the risk of illnesses and stress [35–37,51,52,54,63,68,69].

School characteristics were a significant source of hazard to school teachers. Different schools may present different types and level of hazards in different environments. For example, primary school teachers encounter students with lower levels of cognition and, hence, require more endurance compared to that required for secondary school students. In addition, schools located in urban areas may be more competitive for students’ achievements compared to those in rural areas, which indirectly increases teacher burdens with additional efforts and initiatives to gain better reputations and incentives from the Ministry of Education (MOE).

Besides the limitations already acknowledged, this review had several additional limitations. Initially, it was intended that only articles which obtained full marks on quality assessment during screening be included based on the strength of the study. However, all 22 articles were included for review as the number of studies identified was insufficient for a meaningful analysis. Only one study in this review obtained full marks (7 of 7) in quality assessment while the other 21 articles scored between 2 and 6.

Occupational health research among educators is important as the statistical findings of illnesses and stress may provide insight into their health status. Combined with the health status of educators, the identification of risk factors provides insight and opportunity for relevant authorities, specifically the MOE, to address and mitigate the occupational health problems among educators through policy planning and mechanisms in light of the challenges of Education 4.0 corresponding to technological development and digitization.

## Conclusions and recommendations

In conclusion, despite the lack of study among educators in Malaysia, there is cause for concern as the studies included in this review reported a high prevalence of musculoskeletal disorders, voice disorders, and stress. Besides the commonly reported socio-demographic risk factors (sex, age, education level, body mass index, ethnicity, and religious practice), occupational risk factors (teaching experience, ergonomic issues, workload, and salary), and other risk factors such as personal lifestyle (smoking and alcohol consuming) and psychosocial factors, there were potentially other unexplored contributors to illnesses and stress among educators. By understanding the health status and risk factors of this population, future studies are warranted to explore proper mechanisms and for policy planning to ensure the most appropriate and effective preventive measures.

## Supporting information

S1 Appendix Results of the quality assessment. (DOCX)

S1 Checklist PRISMA 2009 checklist-plos one. (DOCX)

## References

[1] Aguis R. What Is Occupational Health? [Internet]. Occupayional health services 2007 Available from: http://www.agius.com/hew/resource/ohsilo.htm

[2] Karwan MK, Azuhairi AA, Hayati KS. Predictors of Upper Limb Disorders Among a Public University Workers in Malaysia. International Journal of Public Health and Clinical Sciences [Internet]. 2015;2(3):133–50. Available from: http://publichealthmy.org/ejournal/ojs2/index.php/ijphcs/article/view/209

[3] Seifpanahi S, Izadi F, Jamshidi AA, Torabinezhad F, Sarrafzadeh J, Sobhani-Rad D, et al Prevalence of Voice Disorders and Associated Risk Factors in Teachers and Nonteachers in Iran. Journal of Voice [Internet]. 2016;30(4):506.e19-506.e23. Available from: 10.1016/j.jvoice.2015.05.019 26390960

[4] Akinbode R, Lam KBH, Ayres JG, Sadhra S. Voice Disorders in Nigerian Primary School Teachers. Occupational Medicine. 2014;64(5):382–6. 10.1093/occmed/kqu052 24803677

[5] Behlau M, Zambon F, Guerrieri AC, Roy N. Epidemiology of Voice Disorders in Teachers and Nonteachers in Brazil: Prevalence and Adverse Effects. Journal of Voice. 2012;26(5):665.e9-665.e18. 10.1016/j.jvoice.2011.09.010 22516316

[6] Van Houtte E, Claeys S, Wuyts F, Van Lierde K. The Impact of Voice Disorders Among Teachers: Vocal Complaints, Treatment-Seeking Behavior, Knowledge of Vocal Care, and Voice-Related Absenteeism. Journal of Voice [Internet]. 2011;25(5):570–5. Available from: 10.1016/j.jvoice.2010.04.008 20634042

[7] Mohan V, Justine M, Jagannathan M, Aminudin SB, Johari SHB. Preliminary Study of The Patterns and Physical Risk Factors of Work-related Musculoskeletal Disorders among Academicians in A Higher Learning Institute. Journal of Orthopaedic Science [Internet]. 2015;20(2):410–7. Available from: http://www.sciencedirect.com/science/article/pii/S094926581530110X?via=ihub 10.1007/s00776-014-0682-4 25542222

[8] Mohd Anuar NF, Rasdi I, Saliluddin SM, Zainal Abidin E. Work Task and Job Satisfaction Predicting Low Back Pain among Secondary School Teachers in Putrajaya. Iranian Journal of Public Health [Internet]. 2016;45(1):85–92. Available from: http://ijph.tums.ac.ir/index.php/ijph/article/view/6158

[9] Rajan B, Chellappan ME, Thenmozhi. Prevalence of Low Back Pain and its Risk Factors among School Teachers at Bentong, Pahang. International Journal of Physical Education, Sport and Health. 2016;3(2):35–40.

[10] Zamri EN, Moy FM, Hoe VCW. Association of Psychological Distress and Work Psychosocial Factors with Self-reported Musculoskeletal Pain among Secondary School Teachers in Malaysia. Neupane S, editor. PLOS ONE [Internet]. 2017 2 24;12(2):e0172195 Available from: http://dx.plos.org/10.1371/journal.pone.0172195 10.1371/journal.pone.0172195 28234933 PMC5325475

[11] Ariaratnam S, Krishnapillai AD, Daher AM, Fadzil MA, Razali S, Omar SA, et al Relationship between Coping Styles and Lipid Profile among Public University Staff. Lipids in Health and Disease [Internet]. 2017 12 28;16(1):50 Available from: http://lipidworld.biomedcentral.com/articles/10.1186/s12944-017-0438-1 10.1186/s12944-017-0438-1 28245847 PMC5331730

[12] Kovač M, Leskošek B, Hadžić V, Jurak G. Occupational Health Problems Among Slovenian Physical Education Teachers. Kinesiology [Internet]. 2013;45(1):92–100. Available from: https://hrcak.srce.hr/file/153738 10.1080/10803548.2013.11076968 23498704

[13] Claudio L, Rivera GA, Ramirez OF. Association Between Markers of Classroom Environmental Conditions and Teachers’ Respiratory Health. Journal of School Health [Internet]. 2016 6;86(6):444–51. Available from: http://doi.wiley.com/10.1111/josh.12398 27122144 10.1111/josh.12398

[14] Altwigry AM, Almutairi MS, Ahmed M. Gastroesophageal Reflux Disease Prevalence among School Teachers of Saudi Arabia and Its Impact on Their Daily Life Activities. International journal of health sciences [Internet]. 2017;11(2):59–64. Available from: http://www.ncbi.nlm.nih.gov/pubmed/28539865 28539865 PMC5426408

[15] Noor A, Ismail NH. Occupational Stress and Its Associated Factors among Academician in A Research University, Malaysia. Malaysian Journal of Public Health Medicine [Internet]. 2016;16(1):81–91. Available from: https://www.mjphm.org.my/mjphm/index.php?option=com_content&view=article&id=707:occupational-stress-and-its-associated-fac

[16] Ismail N, Abd Rahman A, Zainal Abidin E. Organizational Factors Associated with Occupational Stress among Lecturers in Community Colleges, Peninsular Malaysia. Iranian Journal of Public Health [Internet]. 2014;43(3):125–30. Available from: http://ijph.tums.ac.ir/index.php/ijph/article/view/4889

[17] Makhbul ZM, Sheikh MH, Sheikh K. Measuring the Effect of Commitment on Occupational Stressors and Individual Productivity Ties. Jurnal Pengurusan [Internet]. 2014;40:103–13. Available from: http://ejournals.ukm.my/pengurusan/article/view/7122/2897

[18] Ismail A, Suhaimi FF, Bakar RA, Alam SS. Job Stress with Supervisor’s Social Support as a Determinant of Work Intrusion on Family Conflict. Journal of Industrial Engineering and Management [Internet]. 2013 12 2;6(4):1188–209. Available from: http://www.jiem.org/index.php/jiem/article/view/858

[19] Hamjah SH, Ismail Z, Sham FM, Rasit RM, Ismail A. Spiritual Approach in Managing Work-related Stress of Academicians. Procedia—Social and Behavioral Sciences [Internet]. 2015 2;174:1229–33. Available from: http://linkinghub.elsevier.com/retrieve/pii/S1877042815007934

[20] Ghani MZ, Ahmad AC, Ibrahim S. Stress among Special Education Teachers in Malaysia. Procedia—Social and Behavioral Sciences [Internet]. 2014 2;114:4–13. Available from: http://linkinghub.elsevier.com/retrieve/pii/S1877042813052877

[21] Othman CN, Lamin RAC, Othman N. Occupational Stress Index of Malaysian University Workplace. Procedia—Social and Behavioral Sciences [Internet]. 2014;153:700–10. Available from: http://linkinghub.elsevier.com/retrieve/pii/S1877042814055438

[22] Mukosolu O, Ibrahim F, Rampal L, Ibrahim N. Prevalence of Job Stress and Its Associated Factors among Universiti Putra Malaysia Staff. Malaysian Journal of Medicine and Health Sciences [Internet]. 2015;11(1):27–38. Available from: http://www.medic.upm.edu.my/upload/dokumen/FKUSK1_Article_4_(1).pdf

[23] Yaacob M, Choi SL. Role of Occupational Stress on Job Satisfaction. Mediterranean Journal of Social Sciences [Internet]. 2015;6(2):81–7. Available from: http://www.mcser.org/journal/index.php/mjss/article/view/5867

[24] Azizah A, Rozainee K, Nada I, Izreen S, Norhafizah Z. The Prevalence of Occupational Stress and Its Association With Socio- Demographic Factors Among Lecturers in a Private University in Malaysia. International Journal of Public Health and Clinical Sciences [Internet]. 2016;3(4):63–71. Available from: http://publichealthmy.org/ejournal/ojs2/index.php/ijphcs/article/view/326/270

[25] Chen W, Haniff J, Siau C, Seet W, Loh S, Abd MH. Burnout in Academics: An Empirical Study in Private Universities in Malaysia. The International Journal of Social Sciences and Humanities Invention [Internet]. 2014;1(2):62–72. Available from: https://valleyinternational.net/index.php/theijsshi/article/view/6/6

[26] Achour M, Mohd Nor MR, MohdYusoff MYZ. Islamic Personal Religiosity as a Moderator of Job Strain and Employee’s Well-Being: The Case of Malaysian Academic and Administrative Staff. Journal of Religion and Health [Internet]. 2016;55(4):1300–11. Available from: 10.1007/s10943-015-0050-5 25835985

[27] Wee YG, Bahrein ABBA. Teacher Stress and Workplace Deviance: Does Emotional Intelligence Matter? International Journal of Applied Business and Economic Research [Internet]. 2016;14(13):9283–305. Available from: http://www.serialsjournals.com/serialjournalmanager/pdf/1484118904.pdf

[28] Nor NM, Salleh SHS. Correlation Between Precursor Emotion and Human Stress by Using EEG Signals. Asian Research Publishing Network (APRN) Journal of Engineering and Applied Sciences [Internet]. 2015;10(23):17881–9. Available from: www.arpnjournals.com

[29] Ahmad R, Khan A, Mustaffa MS. Self-Concept and Stress among Junior and Senior School Counselors: A Comparison Case Study in Secondary Schools in Malacca. Mediterranean Journal of Social Sciences [Internet]. 2015 9 1;6(5):593–9. Available from: http://www.mcser.org/journal/index.php/mjss/article/view/7528

[30] Kalyva E. Stress in Greek Primary Schoolteachers Working Under Conditions of Financial Crisis. Europe’s Journal of Psychology [Internet]. 2013 2 28;9(1):104–12. Available from: http://ejop.psychopen.eu/article/view/488

[31] Bogaert I, De Martelaer K, Deforche B, Clarys P, Zinzen E. Associations Between Different Types of Physical Activity and Teachers’ Perceived Mental, Physical, and Work-related Health. BioMed Central (BMC) Public Health [Internet]. 2014 12 30;14(1):534 Available from: http://bmcpublichealth.biomedcentral.com/articles/10.1186/1471-2458-14-534 10.1186/1471-2458-14-534 PMC4066273 24885620

[32] Chong EYL, Chan AHS. Subjective Health Complaints of Teachers From Primary and Secondary Schools in Hong Kong. International Journal of Occupational Safety and Ergonomics [Internet]. 2010 1 8;16(1):23–39. Available from: https://www.tandfonline.com/doi/full/10.1080/10803548.2010.11076825 20331916 10.1080/10803548.2010.11076825

[33] Kovess-Masféty V, Sevilla-Dedieu C, Rios-Seidel C, Nerrière E, Chan Chee C. Do Teachers Have More Health Problems? Results From A French Cross-Sectional Survey. BioMed Central (BMC) Public Health [Internet]. 2006 12 21;6(1):101 Available from: http://bmcpublichealth.biomedcentral.com/articles/10.1186/1471-2458-6-101 10.1186/1471-2458-6-101 PMC1523205 16630336

[34] Ramprasad S, Maruthi YA. Vocal Problem: Neglected Occupational Hazard In Teaching Profession. Sikkim Manipal University (SMU) Medical Journal [Internet]. 2016;3(1):723–35. Available from: http://www.i-scholar.in/index.php/SMU/issue/view/12975

[35] Cheng H-YK, Wong M-T, Yu Y-C, Ju Y-Y. Work-related Musculoskeletal Disorders and Ergonomic Risk Factors in Special Education Teachers and Teacher’s Aides. BioMed Central (BMC) Public Health [Internet]. 2016 2 10;16(1):137 Available from: 10.1186/s12889-016-2777-7 PMC4750223 26864071

[36] Dawn S, Talukdar P, Bhattacharjee S, Singh OP. A Study on Job related Stress among School Teachers in Different Schools of West Bengal, India. Eastern Journal of Psychiatry [Internet]. 2016;19(1):12–7. Available from: http://easternjpsychiatry.org/index.php/about/article/view/49

[37] Ferreira AI, Martinez LF. Presenteeism and Burnout among Teachers in Public and Private Portuguese Elementary Schools. The International Journal of Human Resource Management [Internet]. 2012 11;23(20):4380–90. Available from: http://www.tandfonline.com/doi/abs/10.1080/09585192.2012.667435

[38] Hadi AA, Naing NN, Daud A, Nordin R, Sulong MR. Prevalence and Factors Associated with Stress among Secondary School Teachers in Kota Bharu, Kelantan, Malaysia. Southeast Asian Journal Tropical Medicine and Public Health [Internet]. 2009;40(6):1359–70. Available from: http://www.tm.mahidol.ac.th/seameo/journal-40-6-2009.html 20578472

[39] Huda BZ, Rusli BN, Naing L, Tengku MA, Winn T, Rampal KG. A Study of Job Strain and Dissatisfaction among Lecturers in The School of Medical Sciences Universiti Sains Malaysia. The Southeast Asian journal of tropical medicine and public health [Internet]. 2004 3;35(1):210–8. Available from: http://www.tm.mahidol.ac.th/seameo/journal_35_1_2004.html 15272771

[40] Kaur S. Comparative Study of Occupational Stress among Teachers of Private and Govt. Schools in Relation to their Age, Gender and Teaching Experience. International Journal of Educational Planning & Administration [Internet]. 2011;1(2):2249–3093. Available from: http://www.ripublication.com/ijepa.htm

[41] Seibt R, Spitzer S, Druschke D, Scheuch K, Hinz A. Predictors of Mental Health in Female Teachers. International Journal of Occupational Medicine and Environmental Health [Internet]. 2013 1 1;26(6):856–69. Available from: http://ijomeh.eu/Predictors-of-mental-health-in-female-teachers,2135,0,2.html 10.2478/s13382-013-0161-8 24464565

[42] Offord DR, Kraemer HC. Risk Factors and Prevention. Evidence-Based Mental Health [Internet]. 2000 8 1;3(3):70–1. Available from: http://ebmh.bmj.com/cgi/doi/10.1136/ebmh.3.3.70

[43] Moher D, Shamseer L, Clarke M, Ghersi D, Liberati A, Petticrew M, et al Preferred Reporting Items For Systematic Review and Meta-Analysis Protocols (PRISMA-P) 2015 Statement. Systematic Reviews [Internet]. 2015 12 1;4(1):1 Available from: 10.1186/2046-4053-4-1\nhttp://www.systematicreviewsjournal.com/content/4/1/1\nAll Papers/M/Moher et al. 2015—Preferred reporting items for systematic review and meta-analysis protocols (PRISMA-P) 2015 statement.pdf 25554246 PMC4320440

[44] Parker G, Beresford B, Clarke S, Gridley K, Pitman R, Spiers G. Technical Report for SCIE Research Review on The Prevalence and Incidence of Parental Mental Health Problems and The Detection, Screening and Reporting of Parental Mental Health Problems. [Internet]. Social Policy Research Unit, University of York. 2008. Available from: https://www.york.ac.uk/inst/spru/research/pdf/SCIEReview1.pdf

[45] Sugumaran MNAP, Singh K, Govind S, Wah YC. Study on Prevalence and Risk Factors of Neck Pain Among Aimst University Malaysia Academic Staffs. International Journal of Innovative Technology and Exploring Engineering (IJITEE) [Internet]. 2019;8(5):904–13. Available from: https://www.ijitee.org/wp-content/uploads/papers/v8i5/E3304038519.pdf

[46] Moy FM, Hoe VCW, Hairi NN, Chu AHY, Bulgiba A, Koh D. Determinants and Effects of Voice Disorders among Secondary School Teachers in Peninsular Malaysia Using a Validated Malay Version of VHI-10. PLoS ONE. 2015;10(11):e0141963 10.1371/journal.pone.0141963 26540291 PMC4634998

[47] Roscellalnja HAR. Prevalence of Voice Disorder among Primary School Teachers in Bintulu, Sarawak. Malaysian Journal of Public Health Medicine [Internet]. 2016;16(2):89–98. Available from: https://www.mjphm.org.my/mjphm/index.php?option=com_content&view=category&id=106&Itemid=124

[48] Ng YM, Voo P, Maakip I. Psychosocial factors, depression, and musculoskeletal disorders among teachers. BMC Public Health [Internet]. 2019 12 26;19(1):234 Available from: https://bmcpublichealth.biomedcentral.com/articles/10.1186/s12889-019-6553-3 10.1186/s12889-019-6553-3 30808335 PMC6390562

[49] United Nations. World Economic Situation and Prospects 2018 [Internet]. 2018. 204 p. Available from: https://www.un.org/development/desa/dpad/wp-content/uploads/sites/45/publication/WESP2018_Full_Web-1.pdf

[50] Mohseni Bandpei MA, Ehsani F, Behtash H, Ghanipour M. Occupational Low Back Pain in Primary and High School Teachers: Prevalence and Associated Factors. Journal of Manipulative and Physiological Therapeutics [Internet]. 2014;37(9):702–8. Available from: 10.1016/j.jmpt.2014.09.006 25280458

[51] Yue P, Liu F, Li L. Neck/Shoulder Pain and Low Back Pain among School Teachers in China, Prevalence and Risk Factors. BioMed Central (BMC) Public Health [Internet]. 2012 12 14;12(1):789. Available from: BMC Public Health 10.1186/1471-2458-12-789 PMC3524038 22978655

[52] Erick PN, Smith DR. Low Back Pain among School Teachers in Botswana, Prevalence and Risk Factors. BioMed Central (BMC) Musculoskeletal Disorders [Internet]. 2014 12 30;15(1):359 Available from: http://bmcmusculoskeletdisord.biomedcentral.com/articles/10.1186/1471-2474-15-359 10.1186/1471-2474-15-359 PMC4230345 25358427

[53] Beyen TK, Mengestu MY, Zele YT. Low Back Pain and Associated Factors among Teachers in Gondar Town, North Gondar, Amhara Region, Ethiopia. Occupational Medicine & Health Affairs [Internet]. 2013;01(05):1–8. Available from: http://www.esciencecentral.org/journals/2329-6879/2329-6879-1-127.php?aid=16508

[54] Darwish MA, Al-Zuhair SZ. Musculoskeletal Pain Disorders among Secondary School Saudi Female Teachers. Pain Research and Treatment [Internet]. 2013;2013:1–7. Available from: http://www.hindawi.com/journals/prt/2013/878570/10.1155/2013/878570 PMC3736412 23970968

[55] Abdulmonem A, Hanan A, Elaf A, Haneen T, Jenan A. The Prevalence of Musculoskeletal Pain and Its Associated Factors among Female Saudi School Teachers. Pakistan Journal of Medical Sciences [Internet]. 2014;30(6):1191–6. Available from: http://ovidsp.ovid.com/ovidweb.cgi?T=JS&CSC=Y&NEWS=N&PAGE=fulltext&D=emed13&AN=2015199481%5Cnhttp://sfx.ucl.ac.uk/sfx_local?sid=OVID:embase&id=pmid:&id=doi:10.12669/pjms.306.5778&issn=1682-024X&isbn=&volume=30&issue=6&spage=&pages=&date=2014&title=Pakistan 10.12669/pjms.306.5778 25674106 PMC4320698

[56] Saleem M, Bashir MS, Noor R. Frequency of Musculoskeletal Pain in Female Teachers. Annals Of King Edward Medical University. 2014;20(3):245–51.

[57] Moen BE, Wieslander G, Bakke J V., Norback D. Subjective Health Complaints and Psychosocial Work Environment among University Personnel. Occupational Medicine [Internet]. 2013 1 1;63(1):38–44. Available from: https://academic.oup.com/occmed/article-lookup/doi/10.1093/occmed/kqs188 23144119 10.1093/occmed/kqs188

[58] da Rocha LM, Behlau M, De Mattos Souza LD. Behavioral Dysphonia and Depression in Elementary School Teachers. Journal of Voice [Internet]. 2015;29(6):712–7. Available from: 10.1016/j.jvoice.2014.10.011 26142760

[59] da Rocha LM, de Lima Bach S, do Amaral PL, Behlau M, de Mattos Souza LD. Risk Factors for the Incidence of Perceived Voice Disorders in Elementary and Middle School Teachers. Journal of Voice [Internet]. 2017;31(2):258.e7-258.e12. Available from: 10.1016/j.jvoice.2016.05.018 27427183

[60] Korn GP, Augusto de Lima Pontes A, Abranches D, Augusto de Lima Pontes P. Hoarseness and Risk Factors in University Teachers. Journal of Voice [Internet]. 2015 7;29(4):518.e21-518.e28. Available from: 10.1016/j.jvoice.2014.09.008 25795353

[61] Devadas U, Bellur R, Maruthy S. Prevalence and Risk Factors of Voice Problems Among Primary School Teachers in India. Journal of Voice [Internet]. 2017;31(1):117.e1-117.e10. Available from: 10.1016/j.jvoice.2016.03.006 27363867

[62] Taher YA, Samud AM, Hashemi MM. Prevalence of Depression, Anxiety and Stress Among Libyan Primary and Secondary Schoolteachers: A Cross-Sectional Study. Jordan Journal of Pharmaceutical Sciences [Internet]. 2016 5;9(2):129–40. Available from: https://journals.ju.edu.jo/JJPS/issue/view/487

[63] Ehsani F, Mohseni-Bandpei MA, Fernández-De-Las-Peñas C, Javanshir K. Neck Pain in Iranian School Teachers: Prevalence and Risk Factors. Journal of Bodywork and Movement Therapies [Internet]. 2017 1;22(1):64–8. Available from: http://linkinghub.elsevier.com/retrieve/pii/S1360859217300906 10.1016/j.jbmt.2017.04.003 29332759

[64] Claus M, Kimbel R, Spahn D, Dudenhöffer S, Rose D-M, Letzel S. Prevalence and Influencing Factors of Chronic Back Pain among Staff At Special Schools with Multiple and Severely Handicapped Children in Germany: Results of A Cross-Sectional Study. BioMed Central (BMC) Musculoskeletal Disorders [Internet]. 2014 12 25;15(1):55 Available from: http://bmcmusculoskeletdisord.biomedcentral.com/articles/10.1186/1471-2474-15-55 10.1186/1471-2474-15-55 PMC3996048 24568286

[65] Milczarek M, Schneider E, González ER. OSH in figures: stress at work—facts and figures [Internet]. European Agency for Safety and Health at Work, Office for Official Publications of the European Communities 2009 Available from: https://osha.europa.eu/en/tools-and-publications/publications/osh-figures-stress-work-facts-and-figures/view

[66] Blaak E. Gender Differences in Fat Metabolism. Current Opinion in Clinical Nutrition and Metabolic Care [Internet]. 2001 11;4(6):499–502. Available from: https://insights.ovid.com/crossref?an=00075197-200111000-00006 11706283 10.1097/00075197-200111000-00006

[67] Zadeh NM, Fakhri LS. Primary School Teachers and Occupational Health: Blood Pressure, Voice Hoarseness, Allergy. In: 2011 International Conference on Social Science and Humanity, International Proceedings of Economics Development and Research (IPEDR) [Internet]. 2011. p. 442–5. Available from: http://www.ipedr.com/vol5/no1/94-H00188.pdf

[68] Galgotra M. Mental Health of High School Teachers In Relation To Their Sex and Job Satisfaction. International Journal of Humanities and Social Science Invention [Internet]. 2013;2(1):20–3. Available from: http://www.ijhssi.org/papers/v2(1)/Version-2/D212023.pdf

[69] Jani B. Stress of Teachers Working in Primary Schoool in Kalahandi. International Education and Research Journal [Internet]. 2017;3(1):78–9. Available from: http://ierj.in/journal/index.php/ierj/article/view/658%5Cnhttp://ierj.in/journal/index.php/ierj/article/view/503/475

