# Supplementary material for: Systematic review on the prevalence of illness and stress and their associated risk factors among educators in Malaysia
Source: PLoS One. 2019 May 28;14(5):e0217430. doi: 10.1371/journal.pone.0217430 (PMC6538178; doi:10.1371/journal.pone.0217430)
Supplement: S1 Appendix — (DOCX) [file pone.0217430.s001.docx]

**Appendix 1. Results of the quality assessment**

| **No.** | **Source** | **Year** | **Sample Definition** | **Recruitment** | **Representative Sample** | **Response Rate** | **Scale** | **Sample Size** | **CI/ SE** | **Quality Score** |
| --- | --- | --- | --- | --- | --- | --- | --- | --- | --- | --- |
| 1 | Karwan et al. | 2015 | 1 | 1 | 1 | 1 | 1 | 0 | 0 | 5 |
| 2 | Mohan et al. | 2015 | 1 | 0 | 1 | 0 | 1 | 0 | 0 | 3 |
| 3 | Mohd Anuar et al. | 2016 | 1 | 1 | 0 | 1 | 1 | 0 | 0 | 4 |
| 4 | Rajan et al. | 2016 | 1 | 1 | 0 | 1 | 1 | 0 | 0 | 4 |
| 5 | Zamri et al. | 2017 | 1 | 1 | 1 | 0 | 1 | 1 | 1 | 6 |
| 6 | Sugumaran et al. | 2019 | 1 | 0 | 0 | 1 | 0 | 0 | 0 | 2 |
| 7 | Ng et al. | 2019 | 1 | 0 | 1 | 1 | 1 | 1 | 1 | 6 |
| 8 | Moy et al. | 2015 | 1 | 1 | 1 | 1 | 1 | 1 | 1 | 7 |
| 9 | Roscellalnja | 2016 | 1 | 1 | 0 | 1 | 0 | 0 | 0 | 3 |
| 10 | Ariaratnam et al. | 2017 | 1 | 1 | 0 | 1 | 1 | 0 | 1 | 5 |
| 11 | Azizah et al. | 2016 | 1 | 0 | 1 | 1 | 1 | 0 | 0 | 4 |
| 12 | Chen et al. | 2014 | 1 | 0 | 1 | 1 | 1 | 0 | 0 | 4 |
| 13 | Ismail et al. | 2014 | 1 | 1 | 1 | 1 | 1 | 0 | 0 | 5 |
| 14 | Mukosolu et al. | 2015 | 1 | 1 | 1 | 1 | 1 | 1 | 0 | 6 |
| 15 | Noor & Ismail | 2016 | 1 | 1 | 1 | 1 | 1 | 1 | 0 | 6 |
| 16 | Yaacob& Choi | 2015 | 1 | 0 | 0 | 1 | 1 | 1 | 0 | 4 |
| 17 | Wee & Bahrein | 2016 | 1 | 0 | 1 | 1 | 1 | 1 | 0 | 5 |
| 18 | Nor &Salleh | 2015 | 1 | 0 | 0 | 1 | 1 | 0 | 0 | 3 |
| 19 | Ahmad et al. | 2015 | 1 | 1 | 1 | 1 | 1 | 0 | 0 | 5 |
| 20 | Ismail et al. | 2013 | 1 | 0 | 1 | 0 | 1 | 1 | 0 | 4 |
| 21 | Hamjah et al. | 2015 | 1 | 1 | 1 | 1 | 0 | 0 | 0 | 4 |
| 22 | Ghani et al. | 2014 | 1 | 1 | 0 | 1 | 1 | 0 | 0 | 4 |
